# Whole-Genome Sequencing of *Trypanosoma brucei* Reveals Introgression between Subspecies That Is Associated with Virulence

**DOI:** 10.1128/mBio.00197-13

**Published:** 2013-08-20

**Authors:** Ian Goodhead, Paul Capewell, J. Wendi Bailey, Tanja Beament, Michael Chance, Suzanne Kay, Sarah Forrester, Annette MacLeod, Mark Taylor, Harry Noyes, Neil Hall

**Affiliations:** Centre for Genomic Research, Institute of Integrative Biology, University of Liverpool, Liverpool, United Kingdom^a^; Wellcome Centre for Molecular Parasitology, Institute of Biodiversity Animal Health and Comparative Medicine, University of Glasgow, Glasgow, United Kingdom^b^; Molecular and Biochemical Parasitology, Liverpool School of Tropical Medicine, Liverpool, United Kingdom^c^

## Abstract

Human African trypanosomiasis is caused by two subspecies of *Trypanosoma brucei*. *Trypanosoma brucei rhodesiense* is found in East Africa and frequently causes acute disease, while *Trypanosoma brucei gambiense* is found in West Africa and is associated with chronic disease. Samples taken from a single focus of a Ugandan outbreak of *T*. *b. rhodesiense* in the 1980s were associated with either chronic or acute disease. We sequenced the whole genomes of two of these isolates, which showed that they are genetically distinct from each other. Analysis of single nucleotide polymorphism markers in a panel of 31 Ugandan isolates plus 32 controls revealed a mixture of East African and West African haplotypes, and some of these haplotypes were associated with the different virulence phenotypes. It has been shown recently that *T*. *b. brucei* and *T*. *b. rhodesiense* populations undergo genetic exchange in natural populations. Our analysis showed that these strains from the Ugandan epidemic were intermediate between the reference genome sequences of *T*. *b. gambiense* and *T*. *b. brucei* and contained haplotypes that were present in both subspecies. This suggests that the human-infective subspecies of *T. brucei* are not genetically isolated, and our data are consistent with genomic introgression between East African and West African *T*. *b. brucei* subspecies. This has implications for the control of the parasite, the spread of drug resistance, and understanding the variation in virulence and the emergence of human infectivity.

## IMPORTANCE

We present a genetic study of the acute form of “sleeping sickness” caused by the protozoan parasite *Trypanosoma brucei rhodesiense* from a single outbreak in Uganda. This represents an advance in our understanding of the relationship between the *T*. *b. rhodesiense* and *Trypanosoma brucei gambiense* subspecies that have previously been considered geographically distinct. Our data suggest that introgression of West African-derived *T. brucei* haplotypes may be associated with differences in disease presentation in the East African disease. These findings are not only of scientific interest but also important for parasite control, as they suggest that the human-infective *T. brucei* subspecies are not genetically isolated.

## Introduction

Human African trypanosomiasis (HAT) is a neglected tropical disease caused by two subspecies of the protozoan parasite *Trypanosoma brucei*. Together, these species cause around 10,000 notified cases per annum, although the actual figure is likely to be much greater due to the difficulties associated with monitoring the disease (1). The distribution of the disease is limited only by the presence of its vector, the tsetse fly (*Glossina* subsp.), and by this measure, the disease is endemic in 36 countries in sub-Saharan Africa (2). Two other species of *Trypanosoma*, *T. congolense* and *T. vivax*, cause significant disease in livestock, costing the economies of the affected countries an estimated $1 billion every year (3).

The distinction between the subspecies of *Trypanosoma brucei* is coming under increased scrutiny. Originally proposed by Hoare in 1972, *Trypanosoma brucei gambiense*, *Trypanosoma brucei rhodesiense*, and *Trypanosoma brucei brucei* were distinguished, and classified, by geographical location, clinical presentation, and host range (4). *T*. *b. brucei* infects wildlife and livestock but not humans. *T*. *b. rhodesiense* occurs in southern and southeastern Africa and is associated with acute disease in humans. Its capacity to infect humans is conferred by a single gene: the serum resistance antigen (SRA) gene. *T*. *b. gambiense* is found in West and Central Africa and is observed to cause a chronic disease in humans which often takes months or years to develop into a severe illness. *T*. *b. gambiense* has since been split into two genetically distinct groups; type 1 *T*. *b. gambiense* is clonal (5) and less virulent in experimental rodents than type 2 *T. b. gambiense* (6), while type 2 *T*. *b. gambiense* is more similar to *T*. *b. brucei* (7) and is more infective in experimental rodents than type 1 *T. b. gambiense* (8). There is evidence from microsatellite genotypes and kinetoplast (mitochondrial) DNA sequences of 142 samples species-wide that *T*. *b. rhodesiense* is a host range variant of *T*. *b. brucei* (8, 9).

The distinguishing clinical differences between the two human-infective subspecies are becoming less clear-cut; descriptions of both acute (10) and asymptomatic (11) *T*. *b. gambiense* infections exist from Côte d’Ivoire. In *T*. *b. rhodesiense* there have been asymptomatic carriers in Botswana (12), mild disease in Zambia and Malawi (13, 14), and reports of severe, acute disease in Uganda (15). In the last case, the isolates collected from a 1989 outbreak in southeastern Uganda displayed a correlation between disease profile and the multilocus enzyme electrophoresis (MLEE) strain group, suggesting that genetic variation may underlie the differences in observed virulence. The most prevalent zymodemes from within these strain groups were Busoga 17 (B17) and Zambezi 310 (Z310). Z310 isolates were associated with a more chronic infection than B17 isolates, and patients were often unaware of being infected due to a lack of a chancre at the site of a tsetse bite. Patients infected with Z310 parasites often presented at clinics with the more serious late-stage disease. B17 patients often presented earlier in the course of infection, especially as chancres were often present and patients had learned to associate these with *T*. *b. rhodesiense* infections. Those patients that had been observed with late-stage B17 infections had progressed to this stage rapidly, with severe symptoms.

We have used high-throughput sequencing to identify genome-wide single nucleotide polymorphisms (SNPs) in the genomes of one Zambezi (Z310) strain and one Busoga (B17) strain in order to better understand which genetic loci may contribute to the observed differences in virulence from within a single localized outbreak. We have used SNPs discovered by sequencing to further genotype 31 isolates from Uganda, together with additional controls. We present the first genomic sequence data for the East African form of the disease, which can now be compared to the available *T*. *b. brucei* and *T*. *b. gambiense* sequences. Furthermore, we have compared the genome sequences of the three subspecies, which revealed evidence of genetic exchange between the East and West African populations. We replicated the human clinical phenotype in mouse models. Our data suggest that certain patterns of introgression may be linked with increased-virulence phenotypes.

## RESULTS

### Microsatellite analysis cannot separate the Zambezi and Busoga zymodeme strain groups.

**FIG 1:**
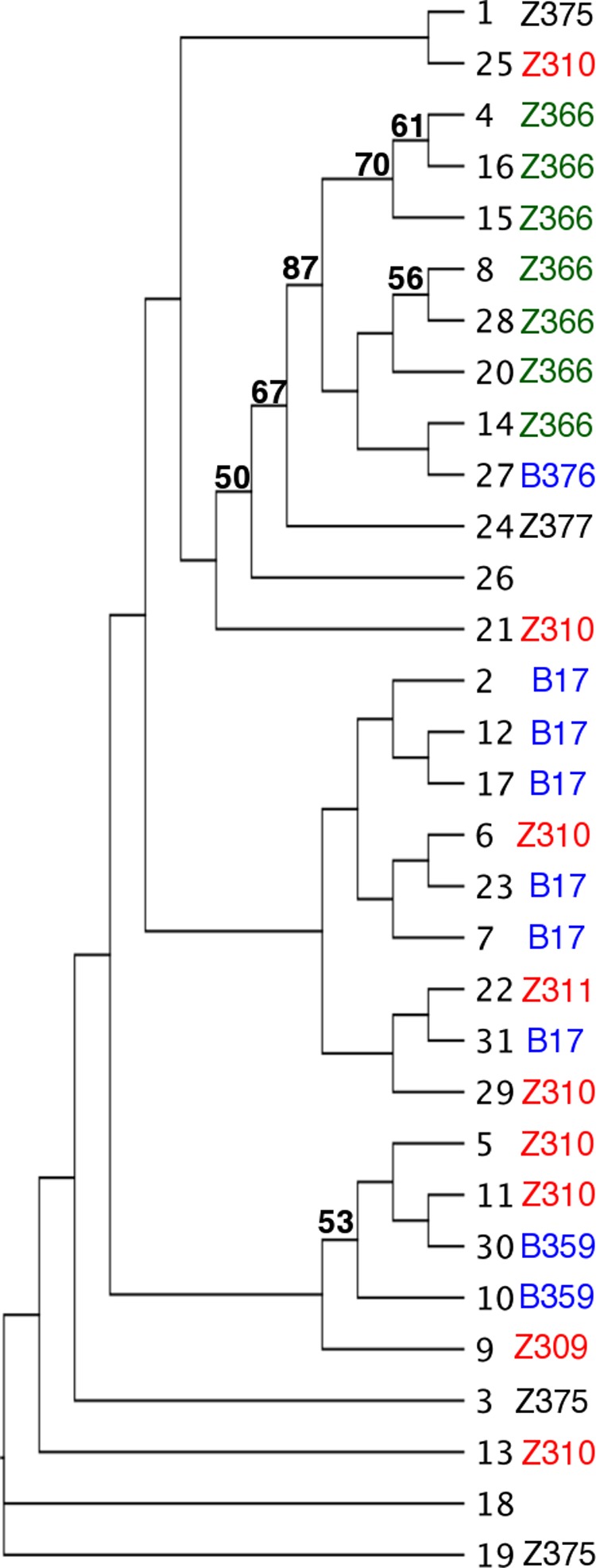
Dendrogram showing the relationship between 31 *T*. *b*. *rhodesiense* isolates at 11 informative microsatellite loci. Isolate numbers (Table S1) are shown alongside their respective zymodemes (where known). The tree was generated using an NJ method using Jaccard’s similarity index. Bootstrap values are based on 100 replicates, and those ≥50 are indicated on the dendrogram. Isolates are color coded according to zymodeme, as follows: Zambezi 366, green; Zambezi 309, 310, and 311, red; Busoga 17 and 376, blue; and Zambezi 375 and 377, black.

Samples originating from the Ugandan outbreak of HAT from 1989 to 1993, for which clinical presentations have previously been described (15) (see Table S1 in the supplemental material), were taken. Thirty-one strains belonging to nine known zymodemes of *T*. *b. rhodesiense* were examined at 11 informative microsatellite loci (Table S2). Clustering the genotype data using a neighbor-joining (NJ) method based on Jaccard’s similarity index revealed few branches with strong bootstrap support. There were three major distinct groups of individuals, including a single group containing Z366 isolates as well as a B376 isolate, a Z377 isolate, and an additional isolate of unknown zymodeme, which clustered separately (Fig. 1).

### Whole-genome sequencing reveals regions of heterozygosity that correspond with shared alleles between *T*. *b. gambiense* and *T*. *b. rhodesiense*.

**FIG 2:**
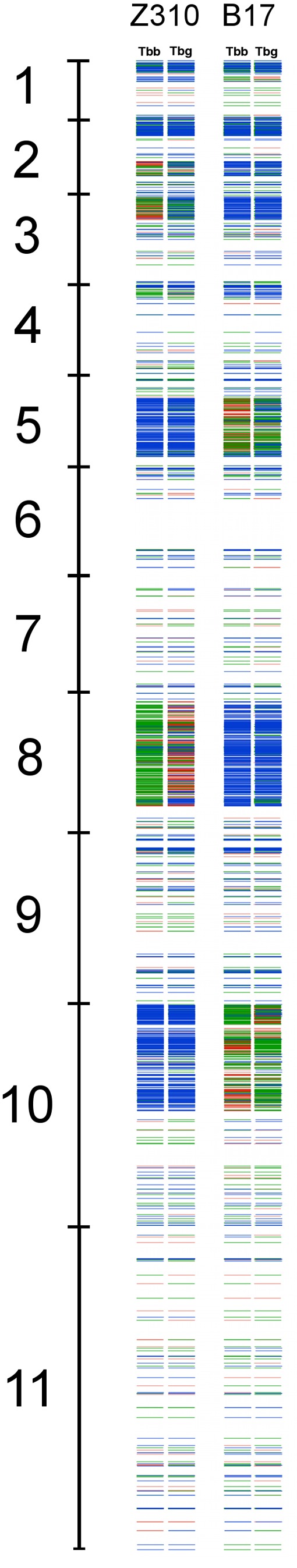
Plot of nonsynonymous SNPs (nsSNPs) in *T*. *b. rhodesiense* Z310 and *T*. *b. rhodesiense* B17 strains. Alleles are shown with respect to their matching allele in either *T*. *b. brucei* TREU927/4 (Tbb) or *T*. *b. gambiense* DAL972 (type 1) (Tbg). In total, 2,787 nsSNPs are displayed (SNPs in VSG-encoding elements have been excluded). Colors are as follows: green, two shared alleles (i.e., the strains are homozygous); blue, one shared allele (i.e., heterozygous); and red, no shared alleles. Chromosomes are represented along the *y* axis. In this manner, Z310 chromosome 8 can be seen to be homozygously similar to *T*. *b. brucei* and not *T*. *b. gambiense*, whereas B17 shares one allele each with *T*. *b. brucei* and *T*. *b. gambiense*.

After sequencing by oligonucleotide ligation and detection (SOLiD), we aligned reads for B17 and Z310 isolates with that of the *T*. *b. brucei* TREU927 reference strain (Table 1). Comparing SNPs of the sequenced *T*. *b. rhodesiense* isolates to both *T*. *b. brucei* TREU927 and type 1 *T*. *b. gambiense* (DAL972) showed regions within chromosomes 2, 3, 5, 8, and 10 in which heterozygosity in one of the sequenced *T*. *b. rhodesiense* isolates was associated with alleles shared with the *T*. *b. gambiense* reference (Fig. 2). The sizes of these regions vary; however, the most striking findings are that ~73% of chromosome 8 of B17 is heterozygous for alleles of *T*. *b. brucei* TREU927 and *T*. *b. gambiense* DAL972 and that Z310 is homozygous and similar to *T*. *b. brucei* TREU927 in the same region, as indicated by a block of blue, representing only one shared allele with the respective reference sequence. Sequence comparison of B17 and Z310 shows that the genomes are >99.8% identical overall; however, at SNP loci, the B17 isolate is 23% more similar to type 1 *T*. *b. gambiense* than the Z310 isolate is.

**TABLE 1:** SOLiD sequencing metrics^*a*^

Zymodeme	Total no. of sequencing reads (thousands)	Mean coverage at SNP loci	No. of SNPs vs *T*. *b. brucei* TREU927/4		No. of SNPs vs alternative *T*. *b. rhodesiense* isolate		
			Total	>10× coverage	Total	Homozygous	Heterozygous
Z310	58.49	53×	132,389	116,065	18,607	4,062	14,545
B17	61.26	52×	137,665	121,202	24,987	6,495	18,492

*^a^* Summary statistics for SOLiD sequencing of two *T*. *b. rhodesiense* strains. Sequencing reads were aligned to the genome and aligned to the ~26-Mbp *T*. *b. brucei* TREU927/4 reference sequence. Mean coverage at SNP loci was determined by the SAMtools (32) Pileup package.

**FIG 3:**
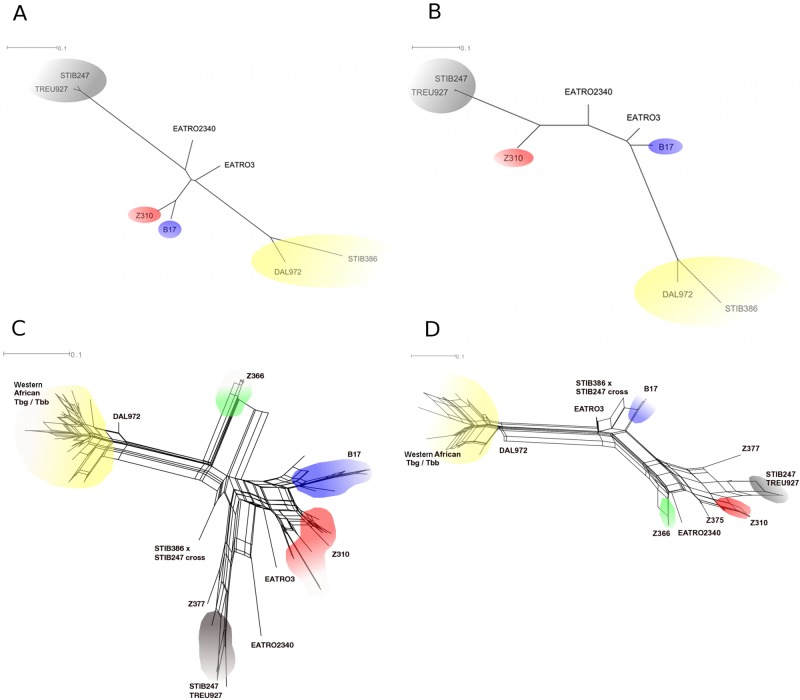
SplitsTree networks and trees. (A and B) Bootstrapped, EqualAngle Jukes-Cantor NJ tree of next-generation whole-genome sequencing data. Bootstraps are based on 1,000 replicates. (A) Genome-wide SNP loci (*n* = 118,161), excluding VSG coding sequences; (B) as in panel A but limited to 9,443 SNP loci on chromosome 8; (C and D) EqualAngle networks for 63 *T. brucei* isolates generated from KASPar genotyping and publicly available next-generation sequencing data; (C) 31 genome-wide SNP loci; (D) 7 SNP loci for chromosome 8 only. Strains included *T*. *b. brucei* TREU927, STIB247, the TREU927 × STIB247 cross, *T*. *b. gambiense* type 1 (DAL972) and type 2 (STIB386), and the *T*. *b. brucei* × *T*. *b. gambiense* cross (STIB247 × STIB386). *T*. *b*. *rhodesiense* strains are indicated by their respective zymodemes, except for EATRO3 and EATRO2340 (see the text in the supplemental material). Colors represent groups of similar subspecies in each zymodeme, as follows: blue, *T*. *b. rhodesiense* B17; red, *T*. *b. rhodesiense* Z310; green, *T*. *b. rhodesiense* Z366; gray, East African *T*. *b. brucei*; and yellow, Central/West African *T*. *b. gambiense*/*T*. *b. brucei*.

A Jukes-Cantor neighbor-joining (NJ) tree of the 118,161 genome-wide SNP loci shows that West African *T*. *b. gambiense* and *T*. *b. brucei* cluster separately from East African *T*. *b. brucei* and *T*. *b. rhodesiense* (Fig. 3A). However, different phylogenetic relationships can be seen upon splitting these data into individual chromosomes. Figure 3B shows a similar Jukes-Cantor NJ tree in which Z310 chromosome 8 is more closely related to chromosome 8 in *T*. *b. brucei* strains than in other strains, whereas *T*. *b. rhodesiense* B17 chromosome 8 is located between chromosomes 8 of East African *T*. *b. brucei* TREU927/STIB247 and West African *T*. *b. gambiense*/*T*. *b. brucei*, as expected, since chromosome 8 is heterozygous for a haplotype that shares one allele with each of *T*. *b. brucei* and *T*. *b. gambiense*.

In order to investigate the position of the *T*. *b. rhodesiense* strains in the context of a wider number of isolates, 50 SNP assays were designed and genotyped using KASPar competitive allele-specific-PCR-based genotyping (details can be found at http://www.kbioscience.co.uk). SNPs were genotyped from 31 *T*. *b. rhodesiense* samples (15) and a single sample from a 2010 patient from Zambia (sample 32) (16). A further 31 isolates from Western Africa (17) representing 5 type 1 and 12 type 2 *T*. *b. gambiense* isolates, 11 *T*. *b. brucei* isolates, and 3 isolates of unknown *T. brucei* subspecies were genotyped in a similar manner (Table S3).

Examining KASPar SNP data on a SplitsTree phylogenetic network revealed patterns consistent with recombination, represented by a net at the center of the tree (Fig. 3C). Interestingly, West African isolates of *T*. *b. brucei* and type 1 and type 2 *T*. *b. gambiense* clustered together, suggesting that West African *T*. *b. brucei* strains are more closely related to *T*. *b. gambiense* than East African parasites of the same subspecies. B17 and Z310 populations are situated together near the other sequenced *T*. *b. rhodesiense* isolates and distinct from East African *T*. *b. brucei* and West African parasites, including *T*. *b. gambiense* and *T*. *b. brucei*. Z366 isolates were tightly clustered, distinct from other *T*. *b. rhodesiense* zymodemes, and situated between *T*. *b. brucei* and *T*. *b. gambiense*. The chromosome 8 SNPs again show that the B17 strains resemble the *T*. *b. gambiense* genotype more closely than the Z310 strains do (Fig. 3D). Figure 3D also shows the B17 zymodeme as equidistant between the East African and West African parasites, which is consistent with it being heterozygous for the two genotypes.

### Human virulence phenotypes can be replicated in experimental mouse infections.

**FIG 4:**
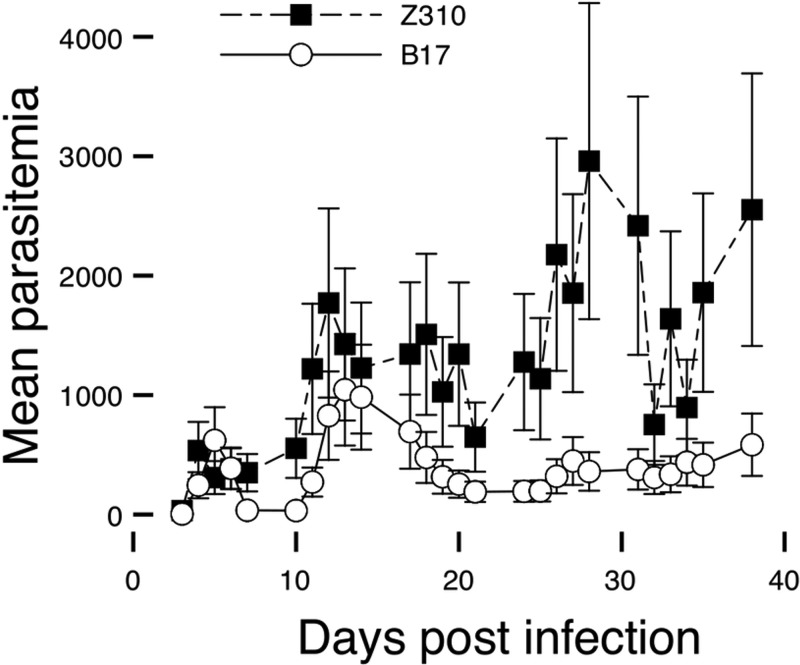
Parasitemia in *T*. *b. rhodesiense*-infected mice. Line graph of mean parasitemia in CD-1 mice infected with B17 and Z310 zymodeme *T*. *b. rhodesiense* parasites ± standard errors (25 fields, thick film, 400× objective). Three isolates of each of the two zymodemes were each used to infect 12 mice (total of 72 mice). We killed 12 mice per week (two per parasite isolate), plus any mice that were killed due to moderate symptoms.

Different clinical phenotypes were observed in humans infected with the two predominant zymodemes from the Ugandan focus of *T*. *b. rhodesiense* (15), and we quantitatively reproduced the different virulence phenotypes in mice. Experimental infections were carried out for three isolates of each zymodeme in outbred CD-1 mice. Parasitemia levels and the overall condition and behavior of the mouse were monitored. Mice infected with B17 isolates survived for longer periods than mice infected with a Z310 isolate despite a higher first peak of parasitemia at 3 to 5 days postinfection (Fig. 4A). B17 isolate-infected mice did not appear ill and never needed to be killed prior to the endpoint of the experiment (38 days postinfection). Z310 parasite-infected CD-1 mice showed a generally higher, more variable level of parasitemia (with a lower initial peak) and showed more-severe symptoms associated with infection; 72% of these mice were humanely killed before the end of the experiment due to deterioration in health. Z310 isolate-infected mice had a significantly higher parasitemia at the time of death than B17 isolate-infected mice (Mann-Whitney, *P* < 0.001). Mice infected with Z310 parasites lived for a significantly shorter length of time than mice infected with B17 trypanosomes (*P* < 0.01).

## DISCUSSION

### Possible origins of the B17 and Z310 genotypes.

Uganda is unique in that populations of all three subspecies of *T. brucei* are present in the country. The known ranges of *T*. *b. gambiense* and *T*. *b. rhodesiense* strains do not overlap (18); however, it is still possible that recombination occurs between subspecies where the ranges of the animal reservoirs of the parasite overlap (e.g., in the underlying *T*. *b. brucei* population). It has been reported recently that microsatellite markers are consistent with recombination between *T*. *b. brucei* and *T*. *b. rhodesiense* (19), yet there have been no reports of recombination in the field between *T*. *b. gambiense* and either of the other subspecies. Our SNP markers suggest that West African *T*. *b. brucei* is more similar to *T*. *b. gambiense* than to East African *T*. *b. brucei*. The B17 and Z310 isolates therefore appear to be the products of recombination between East and West African parasites that have expanded clonally in this particular epidemic. Whether this recombination was directly between *T*. *b. rhodesiense* and *T*. *b. gambiense* or between *T*. *b. rhodesiense* and West African *T*. *b. brucei* cannot be determined from our data. Alternatively, it may be that recombination, followed by losses of heterozygosity at multiple loci, has occurred to generate the different clonal groups.

Other protozoan parasites undergo recombination to produce new, differentially virulent outbreaks; recombination between two ancestral lineages of *Toxoplasma gondii* has resulted in a pandemic outbreak and the currently circulating clonal genotypes. Furthermore, some progeny from laboratory crosses between these lineages were more virulent than the parental lines (20). It remains to be seen whether other species of African trypanosomes also undergo similar processes, although differential virulence is observed within and between different genetic groups of *Trypanosoma congolense* (21). Certainly, the ability for genetic exchange to spread phenotypes, such as virulence or drug resistance, throughout a population means that the situation must be monitored, particularly in the case of *T. congolense* and *T. vivax*, due to the impact of the diseases on human welfare (3).

The reassortment of alleles between different subspecies may have an impact not only on virulence but also on other phenotypes, such as host range and, arguably more importantly, drug resistance; there are increasing reports of treatment failures in the major frontline drugs used in the treatment of sleeping sickness, and resistance is relatively simple to produce in the laboratory (22). Given that, in Uganda, the two forms of HAT exist only 100 km apart (18), these data suggest that close monitoring of the circulating genotypes is necessary if the current trend of decreasing infections across Africa is to continue (1).

### Alleles on chromosome 8 that are shared with West African *T. brucei* may underlie differences in virulence between the B17 and Z310 zymodemes.

Analysis of the genome-wide SNP loci shows that the sampled B17 and Z310 genomes are extremely similar; the mean percentage difference between strains is <0.2%. A heatmap of percentage similarity between the sequenced *T*. *b. rhodesiense* isolates and East African *T*. *b. brucei* TREU927 (Fig. 2A to C) suggests that variation between the B17 and Z310 zymodemes is largely restricted to differences at heterozygous loci on chromosomes 3, 5, 8, and 10. The largest such locus was on chromosome 8, and the B17 isolate is heterozygous across >70% of this 2.5-Mbp chromosome.

The KASPar SNP genotyping and data from public sequence databases for 31 loci across a total of 63 *T. brucei* subsp. isolates from Uganda, Zambia, and Côte d’Ivoire suggest that all isolates within the B17 population have alleles similar to those of the sequenced isolate (see Table S4 in the supplemental material). However, when the entire population of B17 isolates is sampled, there are only a few sites that have a unique allele that is not shared with Z310 isolates. These alleles are clustered on chromosome 8. While there is apparent linkage disequilibrium between these alleles and other parts of the genome, chromosome 8 is a strong candidate for loci that could underlie differences in virulence between B17 and Z310 parasites.

Examining chromosome 8 SNPs on a neighbor-joining tree (Fig. 3B) suggests that B17, EATRO3, and EATRO2340 parasites are heterozygous on chromosome 8, with shared alleles from both type 1 *T*. *b. gambiense* and *T*. *b. brucei* TREU927 (Fig. 3D). The Z310 zymodeme, by contrast, has few shared alleles with *T*. *b. gambiense* DAL972 on chromosome 8 and is more similar to the East African *T*. *b. brucei* TREU927.

Only the isocitrate dehydrogenase (ICD) isoenzyme consistently differentiates between the Zambezi and Busoga strain groups. The ICD gene on chromosome 8 is in the region that is heterozygous for *T*. *b. brucei* TREU927 and type 1 *T*. *b. gambiense* alleles in B17 isolates. The ICD gene contains 3 nonsynonymous SNPs (nsSNPs) that are predicted to modify the charge and isoelectric point of the predicted protein and consequently the mobility of the ICD isoenzyme. ICD mobility determines placement in the Zambezi and Busoga strain groups, which correlates with virulence (15). Therefore, the isoenzyme data are consistent with the SNP genotype data, both of which implicate the heterozygous region of chromosome 8 as a major locus contributing to the observed differences in virulence.

### Conclusions.

Our analysis demonstrates that the *T*. *b. brucei* subspecies causing HAT have undergone genetic exchange in natural populations, since the East African B17 and Z310 parasites share alleles with West African type 1 *T*. *b. gambiense*. The associated differences in disease progression in isolates with differentially derived haplotypes has clear implications for parasite control and diagnosis, as other important traits, such as human serum resistance or drug resistance, may move between parasite groups. It will be important to identify how common this process is and where recombination occurs in the field.

Full-genome sequencing has been able to identify subtle genetic differences between parasite groups that were not apparent from microsatellite typing; however, other polymorphisms, such as small insertions and deletions, could not be detected by the sequencing chemistry. Nevertheless, the SNP data presented in this study have been used to generate a panel of KASPar SNP markers that can now be employed to identify shared alleles and candidate loci underlying phenotypic differences. These techniques could be useful tools for further screening of field isolates in future studies.

## MATERIALS AND METHODS

### Trypanosome stocks.

All parasite samples were from preexisting collections at the Liverpool School of Tropical Medicine and the University of Glasgow and have been described previously (15–17). All parasites were isolated from blood samples collected for diagnostic purposes and collection, and use was not subject to internal review board consent. All samples were anonymized for this study.

All animal work was performed in strict accordance with United Kingdom Home Office regulations under project license number PPL40/3162 (loci controlling the response to *Trypanosoma brucei*).

Thirty-one of the 32 *T*. *b. rhodesiense* isolates used in this study have been previously described (15) (see Table S1 in the supplemental material). Sample 32 was isolated from a patient visiting Zambia in 2010 who presented with a rapidly developing, severe *T*. *b. rhodesiense* infection (16). Samples 33 to 63 are *T*. *b. brucei* and *T*. *b. gambiense* isolates from West Africa from an earlier study (17) Table S3). All of the study isolates had previously been passaged through mice fewer than three times. Zymodeme profiles discussed are according to the work of Stevens et al. (23).

### Multilocus microsatellite genotyping.

Multilocus genotyping at 12 microsatellite loci was used to compare the genotypes of 31 *T*. *b. rhodesiense* isolates. Isolates stored as blood stabilates were recovered from liquid nitrogen, and 0.2 ml was injected intraperitoneally into CD-1 mice. From 3 days postinfection, parasitemia was assessed daily, and those mice found to have a high level of parasitemia were immediately killed and exsanguinated by cardiac puncture; the blood was placed in 4 mM EDTA (used as an anticoagulant). DNA was extracted from mouse blood using a DNeasy blood and tissue kit (Qiagen) per the manufacturer’s instructions. In order to increase the amount of DNA available for PCR and subsequent storage, isolates that had previously been stored as stabilate procyclic cultures were additionally amplified in triplicate using φ29-based whole-genome amplification (Illustra GenomiPhi v2 DNA amplification kit; GE Healthcare), and duplicate reaction mixtures were pooled prior to subsequent use.

Microsatellite repeats on chromosome 8 were identified on the *T*. *b. brucei* TREU927 version 4 (TREU927/4) genome sequence (24, 25) using a local Perl script as previously described (26). PCR primers surrounding these sequences were designed with PRIMER3 (27). Full details of all primers used in this study, 10 of which have been described elsewhere (9, 14), are available in Table S2 in the supplemental material.

PCRs were performed using PCR buffer [45 mM Tris-HCl, pH 8.8, 11 mM (NH_4_)2SO_4_, 4.5 mM MgCl_2_, 6.7 mM 2-mercaptoethanol, 4.4 µM EDTA, 113 µg/ml bovine serum albumin (BSA), 1 mM concentration of each of the four deoxyribonucleotide triphosphates], 1 µM each oligonucleotide primer, and 0.5 U of *Taq* polymerase (custom made by Thermo Scientific) was used per 10-µl reaction mixture. Alternatively, ReddyMix (Thermo Scientific) was used for some PCRs. In both cases, 1 µl of template DNA (20 ng/µliter) was used, except in the case of nested PCR, where 1 µl of a 1/100 dilution of the first product was used in the subsequent nested reaction. The cycling conditions in every case were as follows: 30 cycles of 95°C for 10 s, with a melting temperature (*T*_*m*_) of −5°C for 30 s, and 72°C for 10 s. PCR products were visualized by electrophoresis of ethidium bromide-stained agarose gel (NuSieve GTG; Cambrex, NJ) and visualized under UV light.

Genotyping primers included a 5′-end fluorescent dye modification (6-carboxyfluorescein [FAM]), which enabled accurate detection and sizing using a capillary-based sequencing instrument (ABI 3130/ABI 3100; Applied Biosystems, Foster City, CA, USA) against a set of 6-caboxyl-X-rhodamine (ROX)-labeled proprietary size standards (GS-LIZ500; Applied Biosystems). Allele scores were generated using Peak Scanner software (Applied Biosystems).

A bootstrapped dendrogram showing the relationship between the different *T*. *b. rhodesiense* multilocus genotypes was generated by the neighbor-joining method using Jaccard’s similarity index. One marker that was found to be uninformative across all samples (M12C12) was removed prior to subsequent analysis. Bootstrap values were based on 100 replicates, and those >70 are indicated on the dendrogram (28).

### SOLiD sequencing of B17 and Z310 isolates.

Two isolates of *T*. *b. rhodesiense*, one each of zymodemes B17 (isolate 2) (Table S1) and Z310 (isolate 5), were cultured as described previously (29); briefly, parasites were maintained in SDM-79 supplemented with sterile fetal bovine serum to a concentration of 10% (vol/vol) and streptomycin (10 mg/ml) at 27°C in increasing volumes (2 to 5 ml), until sufficient parasite counts were obtained for DNA extraction and sequencing (~50 ng genomic DNA). DNA from cultured parasites was extracted using a blood and cell culture DNA kit (Qiagen, United Kingdom). Sequencing libraries were prepared and amplified by emulsion PCR according to the manufacturer’s protocols (Life Technologies, USA). Whole-genome sequencing was performed on a single slide using the ABI SOLiD analyzer, version 3 (Life Technologies, Foster City, CA). The resulting colorspace sequences were mapped to the *T*. *b. brucei* TREU927/4 genome sequence (24, 25) using Bowtie (30). Sequencing reads and associated coverage were visualized using the IGV browser (31). SNPs were extracted using the SAMtools pipeline (32) and entered into a MySQL database using a Perl script; SNPs associated with low coverage (<5×) were removed. The resulting filtered SNPs were compared to generate lists of SNPs shared by each isolate and for unique homozygous and heterozygous SNPs for each zymodeme. Nonsynonymous SNPs were identified using a bespoke Perl script.

### Publicly available sequence data.

Publicly available genome sequences for *T. brucei* subsp. isolates were downloaded from the Sequence Read Archive (http://trace.ncbi.nlm.nih.gov) for *T*. *b. brucei* (TREU927/4; accession number ERX009953) and for the progeny of a laboratory cross between *T*. *b. brucei* STIB247 and TREU927 (accession number ERX008996), a 1960 Ugandan *T*. *b. rhodesiense* isolate (EATRO3; accession number ERX007603), a 1977 Kenyan *T*. *b. rhodesiense* isolate (EATRO2340; accession number ERX007601), and the progeny of a laboratory cross between type 2 *T*. *b. gambiense* STIB386 and *T*. *b. brucei* STIB247 (accession number ERX000726). Similarly, sequencing reads for *T*. *b. brucei* (STIB247), a type 1 *T*. *b. gambiense* isolate (DAL972) (33), and a type 2 *T*. *b. gambiense* isolate from the Ivory Coast (STIB386) were downloaded directly from the Wellcome Trust Sanger Institute (WTSI) FTP website (ftp://ftp.sanger.ac.uk/pub/pathogens/Trypanosoma/brucei/T.b.gambiense_sequences/). All publicly available sequence data were generated using an Illumina genetic analyzer, except data for DAL972, which was sequenced using dideoxynucleotide (Sanger) sequencing (33). Sanger sequence read lengths exceed the maximum read length permissible by the Bowtie aligner. Therefore, Sanger reads were artificially split into 50-bp reads using a Perl script and treated as per next-generation sequencing data in order to align all data using the same alignment software. The Illumina genetic analyzer and artificial 50-bp Sanger sequencing reads were aligned to the *T*. *b. brucei* TREU927/4 reference sequence using Bowtie (30). SNPs between the downloaded genomes were extracted using the Pileup feature in the SAMtools package (32). SNP loci were identified where an SNP was present in at least one genome and where there was >5-fold coverage in all genomes studied. For the SOLiD-sequenced genomes of *T*. *b. rhodesiense* B17 and Z310 isolates, the mean coverage at SNP loci was ~52-fold (Table 1). Due to the high copy number and variability in genes coding for variable surface glycoproteins (VSG), any SNP located within the boundaries of a VSG coding sequence were removed. A total of 109,495 non-VSG SNP loci were identified.

### Selection of SNP loci for KASPar genotyping.

In order to genotype a wider range of *T. brucei* subsp. isolates from both Uganda (*T*. *b. rhodesiense*) and West Africa (*T*. *b. brucei*/*T*. *b. gambiense*), duplicate whole-genome amplification (WGA) reactions (Illustra GenomiPhi v2 DNA amplification kit; GE Healthcare) were performed on DNA extracted from stabilates (Ugandan samples) or from Whatman FTA punches (West African samples). DNA was extracted and WGA reactions were performed according to the manufacturer’s instructions as previously described.

Fifty nonsynonymous SNP loci (25 homozygous and 25 heterozygous) were selected from the MySQL database of all SNPs between B17 and Z310 isolates for subsequent typing of the remaining isolates. Loci were evenly distributed across all 11 megabase chromosomes, and all had a low (<0) BLOSUM50 score, indicating that they might modify protein activity. A 100-bp window surrounding the SNPs was extracted from a consensus sequence for the B17 and Z310 genomes using a Perl script and submitted to KBiosciences Ltd. (Hoddesdon, United Kingdom) along with DNA from a total of 63 isolates for SNP genotyping using their proprietary KASPar platform (http://www.kbioscience.co.uk/). Of the 50 SNP loci selected, 31 loci were successfully genotyped (Table S5). These assays are available to other users on application to KBiosciences.

### Genome-wide SNP analyses.

Two thousand seven hundred eighty-seven genome-wide, nonsynonymous SNPs between the *T*. *b. rhodesiense* B17 and Z310 genomes were compared to *T*. *b. brucei* (TREU927/4) and *T*. *b. gambiense* (type 1, DAL972). A plot showing homozygosity and heterozygosity at these loci is shown in Fig. 2. Additionally, a bootstrapped (based on 1,000 replicates) Jukes-Cantor neighbor-joining (NJ) tree was created using SplitsTree for genome-wide SNP loci (Fig. 3A) and for those situated on chromosome 8 (Fig. 3B) (28).

### Nucleotide sequence accession number.

Sequence data have been submitted to the European Nucleotide Archive under study accession number ERP001836.

## SUPPLEMENTAL MATERIAL

Table S1  *T*. *b*. *rhodesiense* isolates used in this study, including details of zymodemes, original storage conditions, and years of collection. Sample 32 was collected from a British tourist visiting Zambia in 2010 (19). All other isolates were collected as previously described (16). Zymodeme profiles discussed are according to the work of Stevens *et al* (23). Table S1, DOCX file, 0.1 MB.

Table S2  Microsatellite loci. Primers used for multilocus microsatellite PCR of 31 Ugandan *T*. *b. rhodesiense* isolates. Forward and reverse primers are listed, along with their chromosome and locus. A fluorescent dye is listed when one was used for capillary-based sequencer sizing. Table S2, DOCX file, 0.1 MB.

Table S3  West African isolates and hosts used in the study. Tbg1, *T. brucei gambiense* type 1; Tbg2, *T. brucei gambiense* type 2; Tbb, *T*. *b. brucei*. Sampling was performed as previously described (20). Table S3, DOCX file, 0 MB.

Table S4  KASPar genotyping and public sequence database data. Genotyping data were obtained by KASPar (KBiosciences Ltd.) and from next-generation sequencing data from public sequence databases. Thirty informative loci are presented for 32 *T. b. rhodesiense* isolates from Uganda, labeled by zymodeme, or “Tbr_unk” if unknown. Highlighted isolates were sequenced by ABI SOLiD. Eight *T. b. brucei/T. b. gambiense* whole-genome sequences deposited in public databases are labeled by strain and laboratory crosses between two isolates are indicated as strain “x” strain (or as a self-fertilized line in the case of TREU927×TREU927). Twenty-three Central/West African *T. b. brucei* and *T. b. gambiense* strains (20) are included and labeled as follows: Tbb = *T. b. brucei*; Tbg1 = *T. b. gambiense* Type 1; Tbg2 = *T. b. gambiense* Type 2; Tb_unk = Unknown subspecies of *T. brucei*. Unknown subspecies of *T. brucei* are indicated with “Tb_unk.” Alleles are color coded as follows: blue, cytosine; black, guanine; red, thymine; green, adenine; and white, unknown. Table S4, XLSX file, 0.1 MB.

Table S5  KASPar-based SNP genotyping loci. A list of SNP loci selected across all 11 megabase chromosomes is shown in order to genotype all *T. b*. *rhodesiense* samples isolated by J. W. Bailey from the 1980s to 1990s Ugandan epidemic. Loci are shown in the context of a 100-bp window with a bracket around the SNP tested, using either the IUPAC code for degenerate bases (e.g., [W]) or each of the individual bases separated by a forward slash (e.g., [A/T]). Table S5, PDF file, 0.1 MB.
