# Supplementary material for: Whole-Genome Sequencing of Trypanosoma brucei Reveals Introgression between Subspecies That Is Associated with Virulence
Source: mBio. 2013 Aug 20;4(4):e00197-13. doi: 10.1128/mBio.00197-13 (PMC3747575; doi:10.1128/mBio.00197-13)
Supplement: Table S1 — T. b. rhodesiense isolates used in this study, including details of zymodemes, original storage conditions, and years of collection. Sample 32 was collected from a British tourist visiting Zambia in 2010 (19). All other isolates were collected as previously described (16). Zymodeme profiles discussed are according to the work of Stevens et al (23). [file mbo004131598st1.docx]

**Supplementary Table 1**

| **Isolate Number** | **Zymodeme** | **Source** | **Disease Stage at Time of Collection** | **Year Collected** |
| --- | --- | --- | --- | --- |
| 1 | **Z375** | Blood | LATE | 1993 |
| 2 | **B17** | Blood | EARLY | 1991 |
| 3 | **Z375** | Blood | EARLY | 1993 |
| 4 | **Z366** | Blood | EARLY | 1992 |
| 5 | **Z310** | Blood | LATE | 1992 |
| 6 | **Z310** | Procyclic | LATE | 1992 |
| 7 | **B17** | Blood | EARLY | 1991 |
| 8 | **Z366** | Blood | EARLY | 1993 |
| 9 | **Z309** | Procyclic | LATE | unknown |
| 10 | **B359** | Blood | LATE | 1992 |
| 11 | **Z310** | Blood | LATE | 1993 |
| 12 | **B17** | Blood | EARLY | 1990 |
| 13 | **Z310** | Blood | LATE | 1993 |
| 14 | **Z366** | Procyclic | EARLY | unknown |
| 15 | **Z366** | Procyclic | EARLY | unknown |
| 16 | **Z366** | Blood | EARLY | 1993 |
| 17 | **B17** | Blood | EARLY | 1991 |
| 18 | **unknown** | Blood | LATE | 1993 |
| 19 | **Z375** | Procyclic | EARLY | unknown |
| 20 | **Z366** | Procyclic | EARLY | unknown |
| 21 | **Z310** | Blood | EARLY | 1990 |
| 22 | **Z311** | Blood | LATE | 1991 |
| 23 | **B17** | Blood | EARLY | 1991 |
| 24 | **Z377** | Blood | LATE | 1991 |
| 25 | **Z310** | Blood | EARLY | 1990 |
| 26 | **unknown** | Blood | EARLY | 1993 |
| 27 | **B376** | Blood | unknown | 1991 |
| 28 | **Z366** | Blood | EARLY | 1993 |
| 29 | **Z310** | Blood | LATE | 1990 |
| 30 | **B359** | Blood | unknown | 1991 |
| 31 | **B17** | Blood | EARLY | 1991 |
| 32 | **unknown** | Blood | EARLY | 2010 |
