# Supplementary material for: Whole-Genome Sequencing of Trypanosoma brucei Reveals Introgression between Subspecies That Is Associated with Virulence
Source: mBio. 2013 Aug 20;4(4):e00197-13. doi: 10.1128/mBio.00197-13 (PMC3747575; doi:10.1128/mBio.00197-13)
Supplement: Table S2 — Microsatellite loci. Primers used for multilocus microsatellite PCR of 31 Ugandan T. b. rhodesiense isolates. Forward and reverse primers are listed, along with their chromosome and locus. A fluorescent dye is listed when one was used for capillary-based sequencer sizing. [file mbo004131598st2.docx]

**Supplementary Table 2**

| **Chromosome** | **Dye** | **Locus** | **FORWARD PRIMER (5’ – 3’)** | **REVERSE PRIMER (5’ – 3’)** |
| --- | --- | --- | --- | --- |
| 1 | FAM | 1/18 inner | tataatgcgtttgtgagaat | tgtgagaatggtactcacgcgctg |
|  |  | 1/18 outer | gaagggagggaacagaagcaggg | caacgttagcacacaattcctgtg |
| 2 | FAM | 2/5 inner | tatcgcggttatgtggatttgtgg | atggcgtgtatcacattcgtgatg |
|  |  | 2/5 outer | cacaacaaaactgccatgaggtac | ccgttggcattaggcacaagta |
| 2 | FAM | PLC inner | ttaagtggacgacgaaataacaaca | caacgacgttggaagagtgtgaac |
|  |  | PLC outer | ttcaaacaccgtccccctcaataat | ccactgacctttcatttgatcgctttc |
| 3 | FAM | 5L5/2 inner | gagcgtacattgcaggtagtgcgtagcg | gtacgtggttaaccacaacctact |
|  |  | 5L5/2 outer | acgaagaaacgaagcaaagaag | ggaaactgcttaaacttgcgtgag |
| 4 | FAM | M12C12 inner | aaaacctcatccagtcgcactgg | tggacacacagaagcctaccg |
|  |  | M12C12 outer | taccctcatcaagtggtcg | agtgtggtggtgcgtgcaaacttgg |
| 5 | FAM | JS2 inner | agtaatgggaatgagcgtcaccag | gattggcgcaacaactttcacatacg |
|  |  | JS2 outer | gatcttcgcttacacaagcggtac | ctttcttccttggccattgttttactat |
| 8 | FAM | Tb8-393863 | CCAGCAGAATGATGCAAAGA | TGGTAGCTTGGCGTCTTACC |
| 8 | FAM | Tb8-1074322 | AGACGAAGCAGCGAGAAGAC | TCTCATTACTGCTCTGTTTTTGC |
| 1 | FAM | Tr401-1 | GTGAAAAACGAAAGGCAACG | TGAGTTCAACAATCTTTTATTCC |
| 2 | FAM | TB2/21 | CTGTGTGTTGCTTGTTCATA | AGTTTAACAGCACTTCCATTT |
| 7 | FAM | Tr407-1 | AACAATATCTGACAATGAGGATGG | GTTTAGATGGGTGGAAAGGGTAGG |
| 11 | FAM | TB11/13 | CAAGAACTCTGCATTGAGC | ATCTGTTGGCGATGGTGA |
