# Supplementary material for: Whole-Genome Sequencing of Trypanosoma brucei Reveals Introgression between Subspecies That Is Associated with Virulence
Source: mBio. 2013 Aug 20;4(4):e00197-13. doi: 10.1128/mBio.00197-13 (PMC3747575; doi:10.1128/mBio.00197-13)
Supplement: Table S3 — West African isolates and hosts used in the study. Tbg1, T. brucei gambiense type 1; Tbg2, T. brucei gambiense type 2; Tbb, T. b. brucei. Sampling was performed as previously described (20). [file mbo004131598st3.docx]

**Supplementary Table 3:**

| **Isolate Number** | **Subspecies** | **Host** |
| --- | --- | --- |
| 33 | Tbg2 | Pig |
| 34 | Tbg1 | Human |
| 35 | Tbb | Dog |
| 36 | Tbg2 | Pig |
| 37 | Tbg2 | Fox |
| 38 | Tbg1 | Pig |
| 39 | Tbg1 | Pig |
| 40 | Tbb | Pig |
| 41 | Tbb | Bushbuck |
| 42 | Tbb | Hartebeest |
| 43 | Tbg1 | Human |
| 44 | Tbg2 | Pig |
| 45 | Tbb | Pig |
| 46 | Tbg2 | Dog |
| 47 | Tbg1 | Kob |
| 48 | Tbb | Pig |
| 49 | Tbb | Pig |
| 50 | Tbb | Dog |
| 51 | Tbb | Pig |
| 52 | Tbg2 | Pig |
| 53 | Tbg2 | Pig |
| 54 | Unknown | Dog |
| 55 | Unknown | Goat |
| 56 | Unknown | Goat |
| 57 | Tbg2 | Pig |
| 58 | Tbg2 | Human |
| 59 | Tbb | Pig |
| 60 | Tbg2 | Pig |
| 61 | Tbb | Pig |
| 62 | Tbg2 | Kob |
| 63 | Tbg2 | Human |
