# Supplementary material for: Whole-Genome Sequencing of Trypanosoma brucei Reveals Introgression between Subspecies That Is Associated with Virulence
Source: mBio. 2013 Aug 20;4(4):e00197-13. doi: 10.1128/mBio.00197-13 (PMC3747575; doi:10.1128/mBio.00197-13)
Supplement: Table S5 — KASPar-based SNP genotyping loci. A list of SNP loci selected across all 11 megabase chromosomes is shown in order to genotype all T. b. rhodesiense samples isolated by J. W. Bailey from the 1980s to 1990s Ugandan epidemic. Loci are shown in the context of a 100-bp window with a bracket around the SNP tested, using either the IUPAC code for degenerate bases (e.g., [W]) or each of the individual bases separated by a forward slash (e.g., [A/T]). [file mbo004131598st5.pdf]

Chromosome Sequence

1 GCTAGGATGAAATAATGAAGTTCGACTGTCAGCGTCATCATCATCATCA[W]CATCAACATCATCAACATCATCGTCATTCTCATCGCGGCAAACGGCAGCA

1 AATGCGAAAGTTGTAACCGTGTTAAAGTGGATTACAAATTTCATTGAAA[C/A]TTTTTTTTGCTGAAGSAAAACATACTGCAACAACAATCKTTACAGCCAAT

1 CACCACCTACGAAAAGCACAGCGCCGCCCAACTGCGCAGCCGAATGCAC[Y]AGCCGMAAACCCCGCCCCGCAGCACAAGACCGGAACCGCGGAGGAAGCGA

2 GCTGCACTTGCGGGACCTTTTCCGACTAAATTCTATGGTGCCATCGTGT[R]GGGTCTTGCCATATCCGGACTGATGACCTCATTTTTGGCTATTGTTATAC

2 CAAGCCGCCTGAATATGTGATCCCGCTTGTTGCCGCAATAACTACCTTG[A]CACTTTGTTCTATTCTGGTAGTAAGTGACGGCAAGCTGGGGATCCTTACC

2 CCGGTCGCAGTCCGTGCCGTTTCTCAGCGTGGCCACGGGGCCGTTTCGT[M]CCTTTTTTCGCTCTCCCTCTTTGGCAAATGACGGCACAGAAGAGATGACT

2 CACAGCTTTTTTTGCCGCTGTGGCTGCTGCTGCTGCCGCTGATGATGAT[R]ATGATAATGATGAGGAAGCCGGTAATTCCCCCAATGTCCCTTTATTGTCT

3 AGTTGCTTAGGTAAAGGTTTGTGAGATTCCGGCACTCGCACCCATTCAA[T/C]CAAACCTCCCTCAGGCTCACACAATCGTTAAGCTCCACTGTCATCAGATT

3 ATTAGCCGCGAAAGCTTGAGTTGGAGCCTCGTTAATATTGAACTCAAAG[K]CAGAGTTCTTATCAGCCTTTGAAATGCAGGTATGCTTCACTGTATGTGAT

3 AAGTTTTTTTGCTGCACGTCTCCAGCACACTAGTTKTTTCCYGGGTTGA[R]GCTCATTATTTCCCATTATATAACGATATTCCCAAGCCACAGAGGGAAAG

3 CGGGGCTCCAGTTTCCTYCATTTTGGTGGTTGTGAGCACATCWGTACTT[Y]CCTTTGCCTTAGACGCGCCTCTCATCCACGAAAACGCACTCGCTACTCGT

4 GCGCACTCTCTCCGCAAGGTCCTCACCCACGGGTTTGCACGATGCCGTA[A/T]GGTGAAGCACACGCAACACATATGTTTGTGCGACAGTTCTCATCCGTGAA

4 TTCTTAGCATATTCTACAGAACTGCATAGTCGTCGTCGCTAACGTGGTG[R]AAAACAATAAGATTRGGATTACTRCTTACGAATGTATGCAGGGGCCTTTG

4 GAAACGTTACAAAGGCGGGCAATTCGTTCAACACATTTTCGAAGCCGCC[R]GGGAAAATGCGAATACTTCTCCTTGCAGTATGATTTAGATGACCATTGAT

4 ACTCCTTTTCTTTTCCCTTGAGAGCGACGGGTGGAAACTCGGCGCACCG[S]TGGCGCCGTAACTTCTTTTCGTGAGCGCCGCCTGCGGTCCTCTACGGTGT

5 GTGTTGTACGCCACCCGTCTGCAGAAGGAGCGCTTTGCCGCAGCGGCTC[C/T]GGACGAAGCGAAGGCGTTACAGGCATTTGAAGCGGCAAAGGCGGATATCG

5 CTCCATTTCCATTTTCTTGATCTTCTTCTTTAGCTTCTTCATTTTCGCC[Y]CGATCTGCTCGGTCTCATCGCGGTGATTTTGTGTGGATTCCTGTAGGCGT

5 GTGGTGGAGATTGTTGRTTCCTTAATGAACTATGTGTTTGTTTATCATA[W]TTTYGATGCGTTTCAGAGCTGCATGATGGGCGTGCTGCGTGGGTGTGGAA

5 GTCTAGTGGAGGCGCCGCTGCTGCTGCTCCTGCCGCCGGTGGCGCACCT[S]CTGCCGCTGCTCCTGCAAAGGAGGAAGAGGAAGATGATGATGATATGGGC

5 ACACGATCGCACCTCACCAGTGACTGAAGCACAAACAAATCGTCGCCGC[S]AACGGAACGTGGGAGCACCACGTTCACTTCTCCATTTGGATATTGACCGC

5 GTTATGCCGCGACAAGCTGCAAATACCTTTCTACCAGTAATTGCTCTAC[M]AGGCGCTASGACAGATGCCCCRTGAGACATTACGGGCAAGACATTACCAA

6 CAGGTAGTTGTCCATGTCATGCTGGAAGTGCTGAATCAGTGAGAACCGA[G/A]TGTCGCTGGCGAGCGGCACTGTTCCAGTTGTGATCAGCTGTCCAGGAGTA

6 ATGATCAGCCTGTTCATCGTTTGGGCTTTTATACAGTTTTCTATCTCCT[G]CCATAAAGATATGTTCTTTTTCTCTTTGTGGGGTTATATTTTCATTTTCA

6 TATTTTCGCGTTCCACGCCCCTGGTATCCTTGTCGTCCGCGGTCCTTTC[M]CCCGCGCCCTCGGCCGTTGTGGCCCCTTCTTTATTATATGGATGAAGTTT

7 GAATTTATCCACCGAATGGCAACAGACAAGATTATCATTTGCTTTTACA[C/T]ACTTATAATGATAGGTATTATTATATTTGCTACGTTAAAGTTTTTGCAAA

7 GCGGCGGCGGGCCGGCGTCTTCTGTGCGAAGGACAACGCCCGCCCCCCA[T/C]GGTGGTCCAGCGGCGTTTTCGCGGGTGATGATTCTCGTGTTAATGGGAGG

7 ATACTGCAAAGGCACCTGTGCAAGSGGCCACCACGTGATAGCGAACTTA[W]TAATCGGTCGGTRACTGAGGTTGAGGGTACTCAGCCCCGTCGTCTTTCAG

8 CTGCGTCGAGTGTGTGATCAGTTGCTTTCWCTCGATCCAGCTAAACGGC[Y]GTCACTGCGACAGCTCTTCCAACAGCCTTTCATTCGCCGTGGCTTAGACT

8 CGGACCTCGTCATTGGTTATATATGATGATGCTCTCAAAAGAACATTCG[G/T]CTCGGGTGCCTCGGCACCCGACAACGAGCCCGCGGATGGGGCGGATGTTT

8 GTTCGAATCACTTACAAGCGCAYGGAAGTGCTCCGCCCGAACGTAAACC[K]GGTCCTTTTCCTTCAGCCTGCTCCGTTCAGACCGTGATTCATCTCCTTGC

8 CTACGCTTTCCGTGAAGAACTTCGACTAAGGCTCCGGATAGAGCAAACT[Y]GTAGTAACACTATGTCGGCTGAGGAGCTGGAAAAACTCAACAAACAGTTT

8 CATCTCATCACCATCCATGTCAACAACCGTCCCACTCACCTTAATGCGA[Y]CMAAGCTTGGACGTGTTGTAGACACAAAGCGCGGTGTTAATGCACCTGCG

8 TCTCATCACCATCCATGTCAACAACCGTCCCACTCACCTTAATGCGAYC[M]AAGCTTGGACGTGTTGTAGACACAAAGCGCGGTGTTAATGCACCTGCGTT

8 TTGGAGCATATGTTGTCGCGCGACTGGGCAGCGCCCCTACGTGGAAGTC[Y]CTCCTTAGGAAAGCGACACGATGCAGTTCAGCAAATTCAGCAAATAACCG

8 TTGGCCCAACTTCAAAGCAGCTCTAACATTACCGCCGCTACGACCAACG[R]AAACGGTGCGACTAATGAGAGCGGATGCGGTGGCTCCACAAGTAGTTCTA

9 TTCCCGTTTTCGCACTCTCTCGTCTCTTTTTCCATAAGCGTGTCCAGCC[A/G]TGCACTTTCATTCGTAAGGTTCCATATCTTACCGTCCTCCTTAGTAACTA

9 GAGGCATATGACAGTCGCGGTCGGCCGAACGCTAAAAAAAAGAAAAACT[T/G]ATCGCCAATAATCTTACATCAACAAGGCGTTTTTCTTTGTGTTTCTTCTG

9 ACCCTTTACGAGATCCCGCGCACGCTCATCAAACCAATTGGGGAATTTA[R]GCCGGCCCGCAAGAATCTTTTCATACGTCCGAATTGGGGTTTCATCAAAA

9 TGGCTACTTTTGACAGTGTTATGCACTGCAGGTATTCGTGCTGACCCAA[W]TAAGGAGGAGTGTCCGGATTTGGGAGGTGATCCATCAGCTGACAACGTAC

10 CAGCAACGCCATGGACGCAAACCCCTGCTCATTACACCTAACGATTAAA[Y]AGACAGGAGGCAGCATCTGCAGCAGACCGACGCCAGCAGGAGCAACGAAA

10 TTTTCACGGAAGTTAAGCAATCTCGGGAGGCAGAGCTTTTGTCTCTATG[R]AACTACTGGGTACAGCTCCGACGTGAAACGCGGGCGCTCTCTGCTGAGGT

10 CTGACGAAAGACCTTGTATTGGGTGATGAATGTAATGGTGCTCATCATC[W]TCTAGACATGACCTTTCCAATACACAATGGCGTCATCCAGAATATGGACG

10 AAGCTTAATGAGGAGTCGTTGGAGCGTTTCTTTGCTATTGTGAACGAGT[Y]CCTTTCGGTTAACCCGWTACAGGTTGAAGCGGTTAGCTTTRCAGTTCATA

10 CCAACTGCCACATCAGCGAATGGTACAGCTCTGCGAGGAGGTCCAACGT[M]CTGAGAATTTTACAGCAGSCGAYCACAACTCCACGGGGTGTCGCGTTGAC

10 GTTCCACAAACGAACTCAACACCAGGTTTTTCAAGTGCCCTCAGCAAAC[R]GGGTACTGTTTTTGGTGGGTGCTGAAGGTCCGCATCCATTACGAGAATGA

10 TGGAAACAATGGCACCACCGTGACGGCCGCTCTTATGGCTCATAAACAC[R]GAGTTTCGTGGCGCACCAAAACTGGAACGAAACAACCGGATTATCTCGGC

11 TTTCCTTTTTGTTGTTTTTTTTTTTTCCCTCCCCCTTCCCCGCCGTTTG[T/A]TGTTGTTTTTTCTTCACTCTTGATTTTATTCCTTTCCTCTCTCTCTCTCT

11 TGCACTCTTTTTTTTTCTGTCTCAAGAAATTCGTTCGCTTCACCTAGTT[T/A]AGCATAAATTTCACGACAAAATTTCATCGCCACCACCACCTCCTCCCTAT

11 GTTCTCCTTAAGGTGGACAACAAACCAGTTTTTGTTCAGTAAATGTACG[R]CGAGAAGTGGGACGAGGTGCTCCCATGGTTGGACGTGGAGCTACATAAGA

11 AAGTRTGTCTTTAATTTTTTTTTGTWTCATCCCTCATCCGTTCACCCGC[M]CAAAGTGTTCCAAGGTGTCGATCATCATGCAGAKGTTGGCCCCTGGTGCT
